# Investigation of Hydrogen and Oxygen Evolution on Cobalt-Nanoparticles-Supported Graphitic Carbon Nitride

**DOI:** 10.3390/ma16175923

**Published:** 2023-08-30

**Authors:** Ausrine Zabielaite, Aldona Balciunaite, Daina Upskuviene, Dijana Simkunaite, Ramunas Levinas, Gediminas Niaura, Jurate Vaiciuniene, Vitalija Jasulaitiene, Loreta Tamasauskaite-Tamasiunaite, Eugenijus Norkus

**Affiliations:** Center for Physical Sciences and Technology (FTMC), LT-10257 Vilnius, Lithuania; aldona.balciunaite@ftmc.lt (A.B.); daina.upskuviene@ftmc.lt (D.U.); dijana.simkunaite@ftmc.lt (D.S.); ramunas.levinas@ftmc.lt (R.L.); gediminas.niaura@ftmc.lt (G.N.); jurate.vaiciuniene@ftmc.lt (J.V.); vitalija.jasulaitiene@ftmc.lt (V.J.); eugenijus.norkus@ftmc.lt (E.N.)

**Keywords:** cobalt, graphitic carbon nitride, annealing, microwave-assisted synthesis, hydrothermal synthesis, hydrogen evolution, oxygen evolution

## Abstract

This study focuses on fabricating cobalt particles deposited on graphitic carbon nitride (Co/gCN) using annealing, microwave-assisted and hydrothermal syntheses, and their employment in hydrogen and oxygen evolution (HER and OER) reactions. Composition, surface morphology, and structure were examined using inductively coupled plasma optical emission spectroscopy, X-ray photoelectron spectroscopy, and X-ray diffraction. The performance of Co-modified gCN composites for the HER and OER were investigated in an alkaline media (1 M KOH). Compared to the metal-free gCN, the modification of gCN with Co enhances the electrocatalytic activity towards the HER and OER. Additionally, thermal annealing of both Co(NO_3_)_2_ and melamine at 520 °C for 4 h results in the preparation of an effective bifunctional Co_3_O_4_/gCN catalyst for the HER with the lower *E*_onset_ of −0.24 V, a small overpotential of −294.1 mV at 10 mA cm^−2^, and a low Tafel slope of −29.6 mV dec^−1^ in a 1.0 M KOH solution and for the OER with the onset overpotential of 286.2 mV and overpotential of 422.3 mV to achieve a current density of 10 mA cm^−2^ with the Tafel slope of 72.8 mV dec^−1^.

## 1. Introduction

Hydrogen demand in 2021 was 94 million tons (Mt), above pre-pandemic levels (91 Mt in 2019), and accounted for about 2.5% of global final energy consumption [1]. Most of the increase was driven by traditional uses in the refining and industrial sectors. However, demand for new applications increased to about 40 Mt (60% more than in 2020). Hydrogen demand is estimated to reach 115 Mt by 2030. This compares with the 130 Mt needed to meet existing climate change commitments made to date by governments around the world and with nearly 200 Mt required by 2030 to achieve net zero emissions by 2050 [1]. In the face of the global energy crisis, the efficient production of hydrogen is becoming one of the mainstays of the hydrogen economy and sustainable development. However, the vast majority of current hydrogen (about 95%) is produced by steam methane reforming and coal gasification processes, which produce CO_2_ and require carbon capture and storage to reduce the environmental impact [2,3]. At the same time, fossil fuels are inevitably consumed. A cleaner and commercially viable alternative is electrochemical hydrogen production through water splitting (WS), although clean hydrogen energy can also be produced from renewable sources (e.g., solar, wind, geothermal, and tidal).

Clean hydrogen production by electrocatalytic WS has recently become a major current priority [4,5,6,7,8]. The electrochemical WS process is based on electrocatalytic reactions, such as the hydrogen evolution reaction (HER) and oxygen evolution reaction (OER), which are critical for the large-scale development of clean energy alternatives. Both reactions require efficient HER/OER electrocatalysts to reduce the activation energy and overpotentials [4,9,10]. Noble metals, such as Pt or Pt-group materials (PGMs, including Pt, Pd, Ru, Ir, and Rh), have been the most widely studied and are currently considered as state-of-the-art for water splitting [11,12,13,14,15,16]. Given the impressively high cost of precious metals and their scarcity in nature, significant progress has been made in the search for alternative, cost-effective, and innovative substitutes. As evidence of this, a rapidly increasing number of publications has been reported on earth-abundant transition/noble metal-free (TMs, M=Co, Ni, Fe, Mn, and Mo) and TMs-based alloys or non-metallic (TMXs, X=N, O, S, C, P, etc.) compounds, aimed to function as active HER/OER electrocatalysts [17,18,19,20,21]. TMXs have received much attention due to their compositions, ease of application for large-scale production, distinctive structural features, abundant active sites, and tunable electronic properties. Co, Ni, and Fe are typically characterized as the most powerful materials for WS [22,23,24,25]. Among them, Co-based electrocatalysts, including cobalt oxides [26], hydroxides [27], phosphides [28], sulfides [29], nitrides [30,31], and selenides [32], play a rather significant role in WS and are widely used in HER or OER [33,34]. However, their catalytic performance and stability do not yet meet the requirements for use in practical applications. Many electrocatalysts based on cobalt suffer from poor electrical conductivity and hence a low charge transfer efficiency [33,34]. Efficient and stable Co-based electrocatalytic materials with sufficient intrinsic electronic structure and unlimited active sites on the surface for optimized WS remain challenging.

Strategies to improve the performance of electrocatalysts are mainly based on increasing the intrinsic activity, the number of exposed active sites, and the charge transfer from the conductive support to the catalyst surface [35,36,37,38]. They are closely supported by different engineering approaches [20,35,36,39,40,41,42,43]. Recently, incorporating Co-based catalytic materials onto suitable substrates, or even growing on 3D substrates, has become a convenient way to improve the active site dispersion and catalytic activity of the catalyst [44,45]. In this regard, carbon-based materials have been the most hotly pursued due to their suitability in various catalytic reactions, high stability, large surface area, ease of processing, and low production costs [46,47]. Coupling transition metal-based materials of different structures and morphologies, such as porous carbon [48], carbon cloth [41], carbon nanofibers [42], carbon paper [43], carbon nanotubes [20], graphitic carbon [44], reduced graphene oxide [45], nitrogen-doped graphene [46], and graphene-based nanostructures [49], has been extensively studied for HER/OER and has proven to be an additional effective strategy to increase the electrocatalytic efficiency by controlling the structural morphology [41,42,43,44,45,46,47,48,49,50].

Among the numerous carbon-based materials, graphitic carbon nitride (g−C_3_N_4_) has been pointed out to have great potential in a variety of photocatalytic and electrocatalytic applications due to its thermal, mechanical, electrical, and tunable physicochemical properties; metal-free nature; non-toxicity; low cost; optical response in the visible light range; ease of preparation; environmental friendliness; biocompatibility; and chemical inertness [48,49,50,51,52,53,54]. Unlike various carbon-based materials, g−C_3_N_4_ is a type of polymeric semiconductor with a graphite-like structure containing a large number of N atoms in the form of specific tri-s-triazine rings, beneficial for catalytic activity. It (g−C_3_N_4_) has high stability due to strong covalent bonds between the carbon and nitrogen atoms and can effectively serve as an electrocatalyst for water splitting, being active for both OER and HER reactions [49,52]. However, low conductivity and limited availability of redox sites limit the applicability of pristine g−C_3_N_4_ [48]. Considerable efforts have been applied to address these shortcomings of g−C_3_N_4_.

Numerous strategies have been employed to enhance the functionality of g−C_3_N_4_, including diverse synthesis methods and conditions that allow obtaining g−C_3_N_4_-based materials in different morphologies and sizes of nanoparticles with the required unique properties for improved catalytic performance. Commonly used synthesis techniques are electrodeposition, physical and chemical vapor deposition, microwave-assisted processes, thermal condensation, hydrothermal and solvothermal synthesis routes, sol-gel processes, thermochemical reactions such as pyrolysis and combustion, etc. [49,51,52,55,56,57]. Thermal condensation approaches enable the degree of condensation to be controlled by determining the formation of either melon or g−C_3_N_4_. Heating the melamine at different temperatures makes it possible to obtain different morphologies of g−C_3_N_4_, ranging from nanosheets to rolled nanosheets, nanotubes, and nanoflakes with nanoparticles, depending on the thermal polymerization temperatures of 500, 520, and 540 °C, respectively [58]. Solvothermal methods for synthesizing g−C_3_N_4_ offer significant advantages, such as the formation of uniform and fine particles, exceptionally high surface area and overall porosity, and low energy consumption [51]. However, they are still time-consuming, requiring several hours to complete particle formation and crystallization. Microwave heating methods provide a wide reaction range and short reaction times suitable for industrial-scale production [59]. Synthesis of g−C_3_N_4_ using this technique for only 30 min led to the formation of submicron spheres and a large surface area of 90 m^2^ g^−1^, with improved photocatalytic efficiency [59]. However, the specimen’s morphology is not easily subject to specific regulation within this method. Hydrothermal syntheses are simple and promising methods that can be used to increase a sample’s crystallinity, crystal size, and shape and obtain a product of high purity and stability [60]. However, they are inconvenient for monitoring the growth process of the sample as the preparation process is heavily dependent on the reaction duration, pH, pressure, temperature, and the type of solvent used.

Beyond this, the functionality of g−C_3_N_4_-based materials can be significantly improved by compounding with conductive counterparts, surface modification, metal/non-metal doping, hybridization, heterostructure formation, etc. [51]. Studies on the bonding of g-C_3_N_4_ to Co-based materials have progressed. Different synthesis approaches have been used. A novel hybrid of Co_3_O_4_ embedded in tubular nanostructures of g−C_3_N_4_ was recently synthesized using a simple chemical method at low temperatures, which showed excellent performance for both OER and HER [61]. The synergistic effect, high surface area, and unique structure made maximum redox sites more easily available for catalysis and provided faster ionic and electronic conduction. The tubular nanostructured hybrid Co_3_O_4_@GCN (graphitic carbon nitride) signified the lowest overpotential of 120 mV (at 10 mA cm^−2^) and a current density of 147 mA cm^−2^ for OER, outperforming the benchmark catalysts of IrO_2_ and RuO_2_. At the same time, it showed a small onset potential of −0.03 V towards HER, very close to the onset potential of commercial Pt/C (−0.01 V). The presence of cobalt, carboxyl/hydroxyl groups, and partially negative nitrogen centers were considered to favor the absorption of the water molecules on the surface, contributing greatly to the initial step of OER and HER. S. Vignesh et al. [62] developed an effective, highly dispersed Co_3_O_4_ growth on a g−C_3_N_4_ surface coupled with quantum dots (QDs) on a ZnS composite via a facile hydrothermal synthesis. It exhibited enhanced HER performance with a small Tafel slope of 71 mV dec^−1^ and a low overpotential of 304 mV at 10 mA cm^−2^. The Co_3_O_4_ and ZnS QDs served as a bridging junction, which functioned as a good electron transfer channel. It provided abundant active sites and effectively prevented the agglomeration of the g−C_3_N_4_ nanosheets during the HER process. Zahra et al. [63] developed a cobalt–nickel sulfide on a g−C_3_N_4_ via a simple hydrothermal synthesis. A three-dimensional nickel foam electrode with a well-defined hierarchical flower-like pattern showed excellent catalytic activity for WS reactions with a low overpotential of 160 mV at 10 mA cm^−2^ for HER and 310 mV at 30 mA cm^−2^ for OER in alkaline media. The authors identified a controllable morphological effect to enhance the electrochemical performance and showed that optimized g−C_3_N_4_ concentration plays a vital role in inducing the formation of hierarchical flower-like patterns, which not only prevents agglomeration but also provides a porous structure for enhanced gas diffusivity and charge transfer rates due to the synergistic carbon and nitrogen bonding with Co and NiS. Y. Shen et al. [64] prepared g−C_3_N_4_@Co(OH)_2_ nanowires using a plasma modification strategy. The developed catalyst was subjected to a 60 s plasma treatment, which generated more exposed active edge sites and increased the number of Co^3+^ active sites for enhanced OER activity, displaying an overpotential of 329 mV. B. Shalini Reghunath et al. [65] prepared an effective cobalt ferrite/graphitic carbon nitride/N doped graphene QDs electrocatalyst using hydrothermal synthesis. Notably, this electrocatalyst demonstrated a very low overpotential towards HER of 287 mV at 10 mA cm^−2^ with a Tafel slope of 94 mV dec^−1^ and an overpotential towards OER of 445 mV at 10 mA cm^−2^ with a Tafel slope of 69 mV dec^−1^. S.Y. Ejeta et al. [66] presented Co-incorporated graphitic carbon nitride as a bifunctional catalyst for electrochemical WS in acidic media. The g−C_3_N_4_ powder was synthesized using pyrolysis of melamine and exfoliation via strong sonication, while the reduction of the Co precursor with sodium borohydride synthesized the composite of Co on g−C_3_N_4_. It showed excellent catalytic activity for WS reactions with a minimum overpotential of ~85 mV and ~530 mV for HER and OER, respectively, at 10 mA cm^−2^. It also had a current density of 10 mA cm^−2^ at a cell voltage of 1.84 V, slightly lower than the benchmark catalyst (Pt/Cp).

Various strategies for synthesizing high-performance transition metal-modified g-C_3_N_4_-based catalysts, including annealing, microwave-assisted, and hydrothermal approaches, have been the subject of considerable research interest [62,63,64,65,66]. Each strategy has its own advantages and disadvantages. For example, thermal annealing is inexpensive, simple, and easy to operate [67,68]. However, it is often associated with aggregation phenomena and produces a low surface area catalyst, which limits its use for WS. Hydrothermal synthesis can produce crystallized nanoparticles of the desired shape and size with enhanced chemical activity [62,63]. But the synthesis is carried out under high pressure and requires expensive autoclaves. It is difficult to control the synthesis process and raises safety concerns. Microwave-assisted synthesis is an advantageous, highly effective, fast, and environmentally friendly process. It is often combined with other methods and allows the production of nanostructures of various shapes and sizes [66,69,70]. In addition, uniform microwave heating results in a homogeneous, uniform particle size distribution. However, it depends on microwave heating frequencies and therefore carries the risk of producing an impure synthesised compound.

Although many review articles have been published on the synthesis and application of modified g-C_3_N_4_-based materials for HER and OER, the state of the art in research is not fully understood and explored. The modification of g-C_3_N_4_ with a co-catalyst is achieved by various means and synthesis methods that determine the structure of the catalyst, the particle size and distribution, and thus the catalytic performance. Controlling and combining the relevant preparation methods and approaches can yield g−C_3_N_4_ with different morphologies and unique physicochemical properties required for various applications. However, correlations between the various synthesis methods (for a given catalyst and a given catalytic process) have rarely been reported [70,71].

In this study, different preparation methods—thermal annealing, microwave-assisted, and hydrothermal methods—were used to synthesize Co-supported g-C_3_N_4_. The obtained catalysts were characterized in terms of composition, structure, and catalytic activities. It was shown that thermal annealing is a cost-effective and simple approach for preparing the bifunctional Co_3_O_4_/gC_3_N_4_ catalyst for its applications in electrochemical WS.

## 2. Materials and Methods

### 2.1. Materials and Synthesis

Materials involved included melamine (99%), Co(II) nitrate (Co(NO_3_)_2_, 99%), Co(II) acetylacetonate (C_10_H_14_CoO_4_, 99%), Co(II) chloride (CoCl_2_·6H_2_O, 98%), sodium chloride (NaCl, 98%), tetraethylene glycol (TEG, HO(CH_2_CH_2_O)_3_CH_2_CH_2_OH, 99%), hexamethylenetetramine (HMT, 99–100.5%), sodium hydroxide (NaOH, 98.8%), ethanol (C_2_H_5_OH, 96%), and 5 wt.% Nafion were purchased from Merck KGaA supplier (Darmstadt, Germany).

The gCN was prepared using thermal annealing of melamine at 520 °C for 4 h in a closed high-alumina crucible. A 5 °C/min rate was used to obtain the 520 °C temperature. After synthesis, it was ground into a fine powder.

Co-supported gCN catalysts were prepared using three different methods. One was a thermal treatment of both Co(NO_3_)_2_ and melamine at 520 °C for 4 h, with the resulting catalyst denoted as Co_3_O_4_/gCN. The other two were microwave (MWS) and hydrothermal (HTS) syntheses, which resulted in the preparation of catalysts denoted as Co/gCN-MWS and Co/gCN-HTS, respectively. Briefly, for MWS, a reaction mixture containing 30 mg of synthesized gCN, 14.55 mg of Co(II) acetylacetonate, and 8 mL of TEG was placed in the microwave reactor Monowave 300 (Anton Paar, Graz, Austria). The synthesis was carried out at 270 °C for 1 h. After synthesis, the obtained catalyst was filtered, washed with ethanol and ultra-pure water with a resistivity of 18.2 MΩ cm^−1^, and then dried in a vacuum oven at 80 °C for 2 h.

For the hydrothermal synthesis, 150 mg of CoCl_2_·6H_2_O, 600 mg of HMT, and 375 mg of NaCl were dissolved in 30 mL of water and ethanol solution (the volume ratio being 1:5). The reaction mixture was stirred for 15 min. Then, 40 mg of gCN was added to the mixture and stirred for 4 h. The synthesis was carried out at 120 °C for 8 h in an autoclave.

### 2.2. Materials’ Characterization

The crystallinity of studied catalysts was measured using an X-ray diffractometer D2 PHASER (Bruker, Karlsruhe, Germany). The XRD patterns were recorded in the 2θ range 10–90°.

X-ray photoelectron spectroscopy (XPS) was used for the samples’ chemical characterization using Kratos AXIS Supra+ spectrometer (Kratos Analytical, UK, Manchester, 2019) with the monochromatic Al Kα (1486.6 eV) X-ray radiation powered at 225 W. A low electron flood gun was used as a charge neutralizer. The base pressure in the analysis chamber was less than 1 × 10^−8^ mbar. The survey spectra for each sample were recorded at a pass energy of 80 eV with a 1 eV energy step and high-resolution spectra (pass energy—10 eV, in 0.1 eV steps) over individual element peaks. The binding energy scale was calibrated by setting the adventitious carbon peak at 284.8 eV. Avantage software (Version V5, Thermo Scientific, East Grinstead, UK) was used for the conversion of XPS data to VAMAS format and their processing.

Raman spectra were recorded using in Via Raman (Renishaw, Wotton-under Edge, UK) spectrometer equipped with a thermoelectrically cooled (−70 °C) CCD camera and microscope. Raman spectra were excited with 830 nm radiation from a diode laser (Renishaw, Wotton-under Edge, UK). The 20×/0.40 NA objective lens and 830 lines/mm grating were used to record the Raman spectra. The accumulation time was 200 s. To avoid damage of the sample, the laser power at the sample was restricted to 1.7 mW. The Raman frequencies were calibrated using the polystyrene standard. The spectra were background-corrected using a 6-order function fit. Parameters of the bands were determined by fitting the experimental spectra with Gaussian and Lorentzian shape components using GRAMS/A1 8.0 (Thermo Scientific, Waltham, MA, USA) software

The composition of the catalyst was analyzed using the spectrometer Optima 7000DV (Perkin Elmer, Waltham, MA, USA).

### 2.3. Electrochemical Measurements

The experiments were performed using a potentiostat/galvanostat PGSTAT100 (Metrohm Autolab B. V., Utrecht, The Netherlands). The three-electrode standard cell was used, where a glassy carbon (GC) electrode modified with the synthesized catalysts ink was used as the working electrode. The geometric surface area of the GC electrode was 0.196 cm^2^ as the counter and reference electrodes employed an Ag/AgCl (3 M KCl) and GC road, respectively. Linear sweep voltammograms (LSVs) were recorded in an N_2_-saturated 1 M KOH solution at a scan rate of 2 mV s^−1^. All reported potential values were referred to as “*E*_RHE_”—reversible hydrogen electrode according to Equation (1):

*E*_RHE_ = *E*_measured_ + 0.059·pH + *E*_Ag/AgCl (3 M KCl)_ (1)

where *E*_Ag/AgCl (3 M KCl)_ = 0.210 V.

Current densities for HER and OER presented in this paper were normalized to the geometric area of catalysts.

The catalyst’s ink was obtained by mixing 10 mg of the synthesized catalysts with 1980 μL deionized water and ethanol solution ultrasonically for 2 h, with the volume ratio being 1:1 and 20 μL 5wt.% Nafion solution. Then, 10 μL of the obtained ink was dripped on a pre-prepared GC electrode and dried at 80 °C for 4 h.

Electrochemical impedance spectroscopy (EIS) experiments were carried out on a Zahner Zennium electrochemical workstation (Zahner-elektrik, Kronach-Gundelsdorf, Germany). The spectra were obtained potentiostatically in a deaerated 1 M KOH solution using a 10 mV potential perturbation amplitude and in the frequency region from 10 kHz to 0.1 Hz.

For the determination of the electrochemically active surface area (ECSA) of catalysts, the double layer capacitance (C_dl_) was determined by recording CV curves at various scan rates under the non-faradaic region followed by the calculation of the slope of the curve obtained by plotting the difference in anodic and cathodic current against the scan rate [72,73,74]. From the CVs, the charging current, *I*_c_, of the electrodes at each scan rate was determined via Equation (2):

*I*_c_ [A] = (*I*_anodic_−*I*_cathodic_)_OCP_ (2)

C_dl_ values were evaluated by plotting a graph of charging current vs. scan rate and calculating the slope, as shown by Equation (3):

Slope = C_dl_ [F] = Δ*I*_C_ [A]/Δν [V s^−1^] (3)

Then, the ECSA values were calculated using the specific capacitance (C_s_) of 40 μF cm^−2^ [72,73,74] and Equation (4):

ECSA [cm^2^] = C_dl_ [μF]/C_s_ [μF cm^−2^] (4)

The stability test of the Co_3_O_4_/gCN catalyst was carried out for 1000 cycles at a constant scan rate of 100 mV s^−1^ at the rotation speed of 1600 rpm, after which the stable polarization curve was recorded at 2 mV s^−1^ for comparison with the initial curve.

## 3. Results and Discussion

### 3.1. Characterization of Catalysts

**Figure 1:**
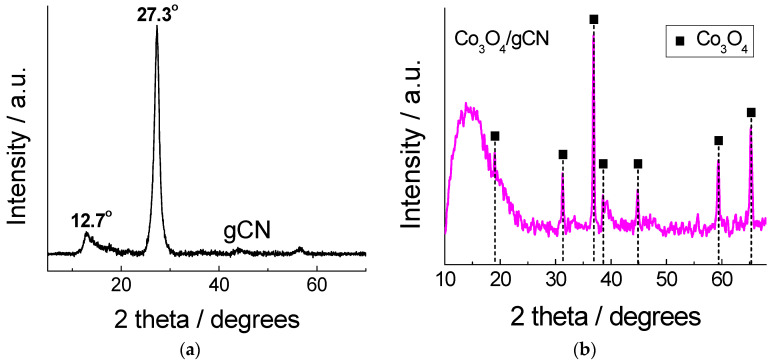
XRD patterns for (**a**) gCN and (**b**) Co_3_O_4_/gCN.

The XRD pattern of as-prepared gCN exhibited a typical pattern, with two broad peaks centered approximately at 12.7° and 27.3° (Figure 1a), and can be assigned to the (100) and (002), respectively, planes of the trigonal N bond of tri-s-triazine and the layered packing of conjugated aromatic units in g−C_3_N_4_, respectively [58,59,60,75]. Figure 1b presents the XRD pattern for Co_3_O_4_/gCN obtained by annealing Co(NO_3_)_2_ and melamine at 520 °C for 4 h. The profile shows the diffraction peaks obtained at 2θ = 18.991, 31.308, 36.848, 38.587, 44.816, 59.379, and 65.238° attributed to crystallographic planes (111), (220), (311), (222), (400), (511), and (440), respectively, of the Co_3_O_4_ phase (ICDD # 01-078-1969). This means that Co_3_O_4_ can be readily obtained using a simple one-step annealing procedure with high crystallinity and without any impurities, confirming the high purity of the prepared Co_3_O_4_.

**Figure 2:**
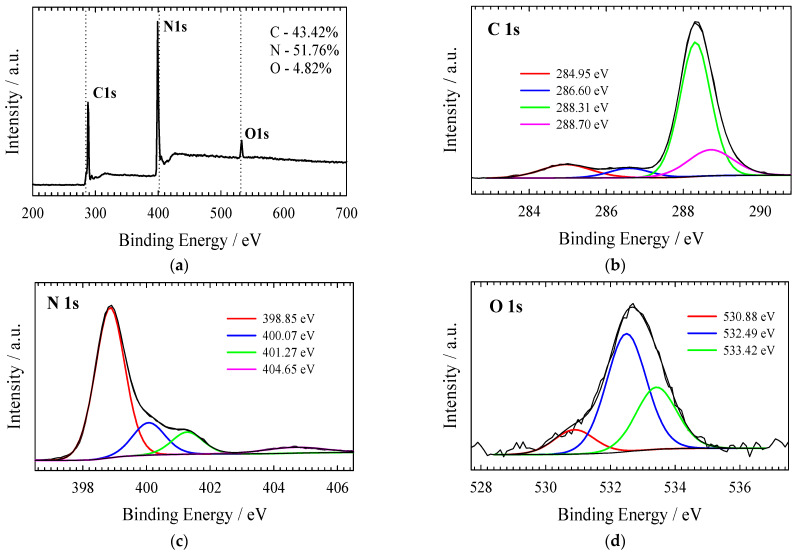
(**a**) The survey and high-resolution XPS spectra of (**b**) C 1 s, (**c**) N 1 s, and (**d**) O 1 s for gCN obtained by annealing the melamine at 520 °C for 4 h.

**Figure 3:**
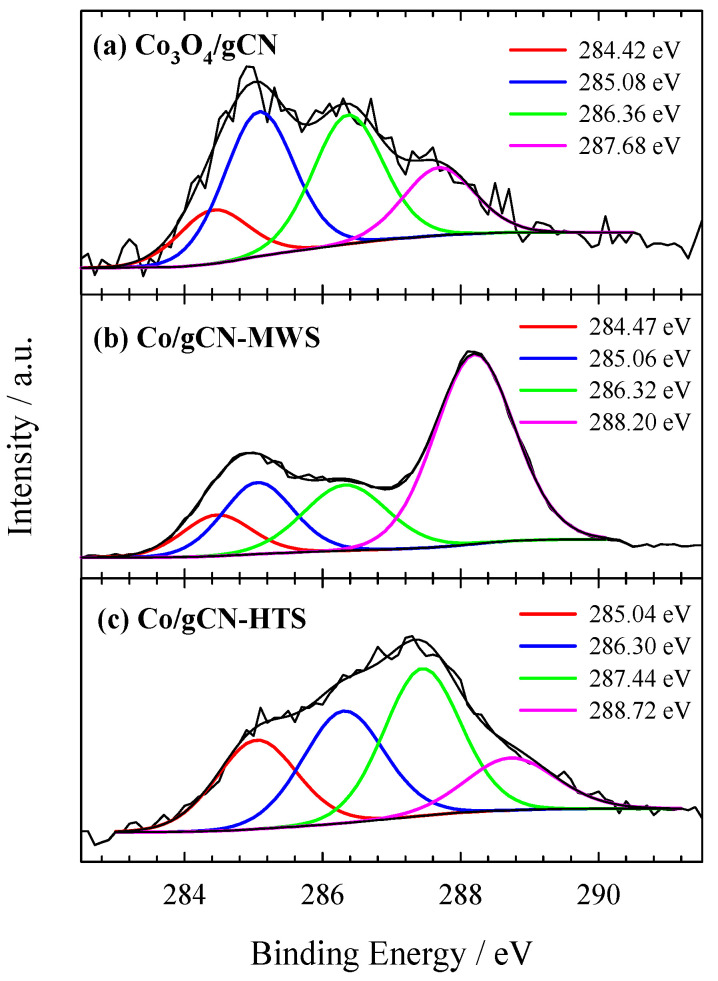
High-resolution XPS spectra of C 1s for (**a**) Co_3_O_4_/gCN, (**b**) Co/gCN-MWS, and (**c**) Co/gCN-HTS.

**Figure 4:**
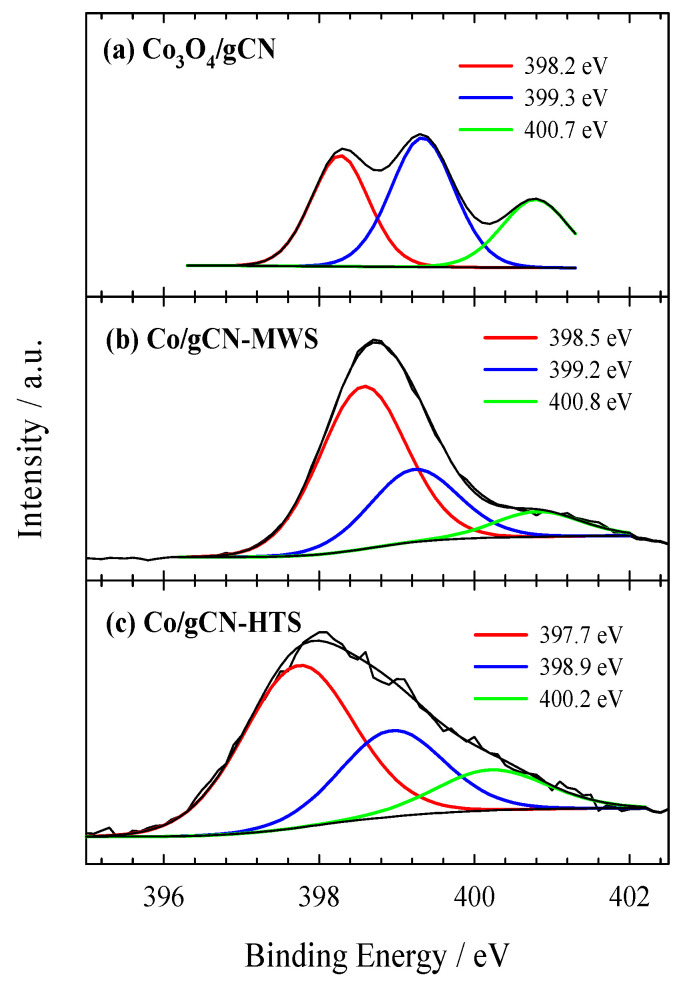
High-resolution XPS spectra of N 1s for (**a**) Co_3_O_4_/gCN, (**b**) Co/gCN-MWS, and (**c**) Co/gCN-HTS.

**Figure 5:**
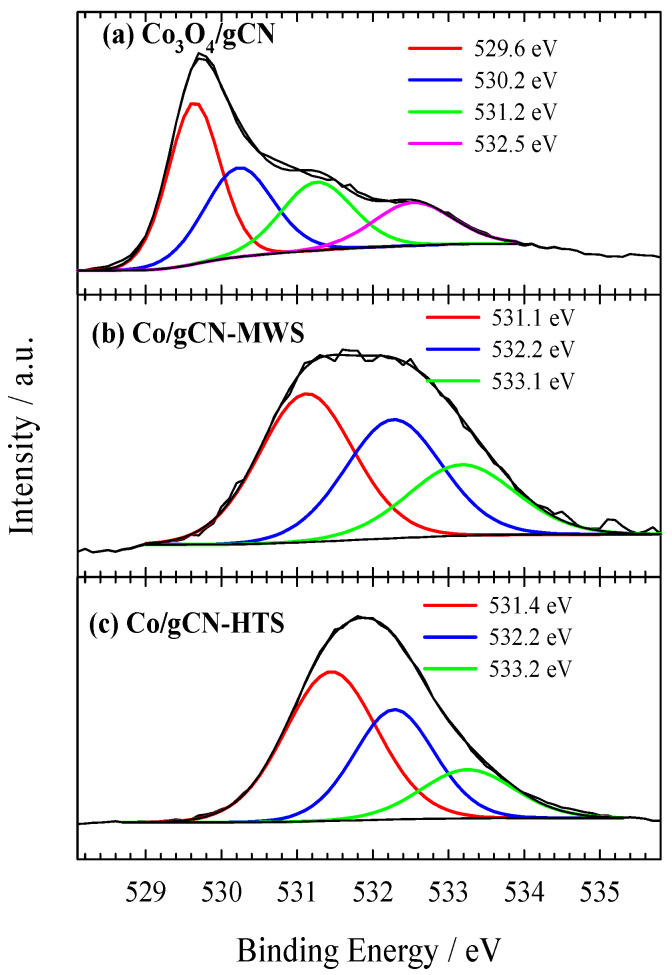
High-resolution XPS spectra of O 1s for (**a**) Co_3_O_4_/gCN, (**b**) Co/gCN-MWS, and (**c**) Co/gCN-HTS.

**Figure 6:**
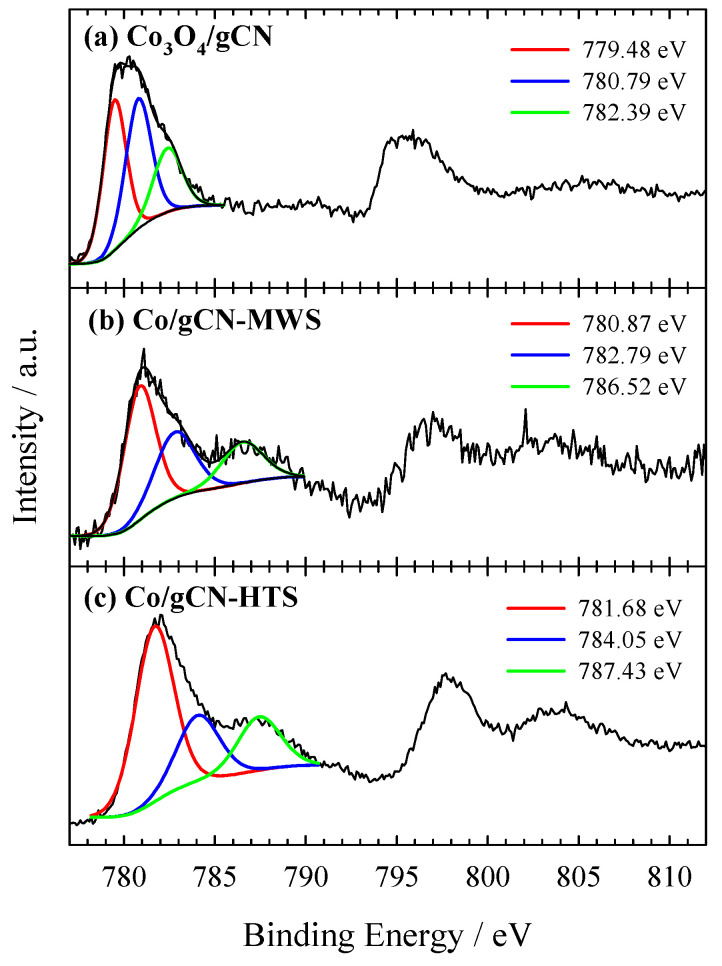
High-resolution XPS spectra of Co 2p for (**a**) Co_3_O_4_/gCN, (**b**) Co/gCN-MWS, and (**c**) Co/gCN-HTS.

XPS was used to investigate the electronic structure and surface composition of the as-prepared gCN, Co_3_O_4_/gCN, Co/gCN-MWS, and Co/gCN-HTS catalysts. XPS analysis data on the elemental composition of the investigated catalysts are given in Table 1, whereas XPS spectra for C 1s, N 1s, O 1s, and Co 2p are presented in Figure 2, Figure 3, Figure 4, Figure 5 and Figure 6.

The survey scan of the XPS for the pristine g-C_3_N_4_ shows that the synthesized material is mainly composed of carbon and nitrogen, with the weakly pronounced O1s peak (Figure 2a) possibly originating from adsorbed oxygen. The high-resolution XPS of the C 1s spectrum (Figure 2b) for the g-C_3_N_4_ shows four peaks centered at around 284.95 eV, 286.60 eV, 288.31 eV, and 288.70 eV. The strongest peak at 288.31 eV corresponds to sp^2^ bonded carbon (N−C=N), and the weaker one at 284.95 eV is attributed to graphitic carbon, which is typically found in the XPS spectrum characterizing g-C_3_N_4_ [76]. The high-resolution XPS of the N 1s spectrum (Figure 2c) can be deconvoluted into four peaks located at 398.85 eV, 400.07 eV, 401.27 eV, and 404.65 eV that can be attributed to sp^2^-hybridized nitrogen (C–N=C), tertiary nitrogen N–(C)_3_ groups, amino functions caring hydrogen (C–N–H) and π-excitation, respectively [58,76,77]. O 1s signals at 530.89 eV, 532.49 eV, and 533.42 eV in Figure 2d are attributed to the oxygen of O=C, O-C, and OH, respectively [76]. Similarly, from the high-resolution C 1s spectrum of oxidized g–C_3_N_4_, two new C1s peaks at 286.60 and 288.70 eV can be deconvoluted, which are attributed to the carbon of O-C and O=C-O, respectively [76].

The XPS spectra show differences in both shape and peak position depending on the catalyst preparation conditions. The high-resolution XPS of the C 1s spectrum (Figure 3a) for the Co_3_O_4_/gCN catalyst can be resolved into several peaks centered at around 284.42, 285.08, 286.36, and 287.68 eV. This confirmed the existence of the g–C_3_N_4_ phase corresponded to adventitious sp^2^ C–C carbon species (284.42 eV), sp^2^ hybridized carbon atoms in N–C–N (286.38 eV), and sp^2^ C=N bond (287.36 eV) in the s-triazine ring, respectively [58,59,78]. In addition, carbon at 285.08 eV and 287.68 eV can be resolved into C–O and CO=, respectively, indicating a strong interaction between g–C_3_N_4_ and Co_3_O_4_ [78].

The N 1s signal shown in Figure 4a is located in the binding energy region of 398–401 eV and can be fitted into three peaks located at 398.27 eV, 399.33 eV, and 400.78 eV for sp^2^-hybridized nitrogen in triazine rings (C–N=C), tertiary nitrogen N–(C)_3_ groups, amino functions caring hydrogen (C–N–H), respectively [58,78]. Furthermore, the peak at 399.33 eV is prevailing, accounting for 42.74 at.%. This binding energy area covers the binding energy region required for the N−Co bond, which is difficult to distinguish [64,77,79]. The binding energy of pyridine N in the Co_3_O_4_/g−CN sample (398.27 eV) decreased by 0.58 eV compared with that of pristine in g−C_3_N_4_ (398.85 eV), reflecting the interfacial interaction between g–C_3_N_4_ and Co_3_O_4_.

O 1s signals in Figure 5a at 529.64 eV (36.81 at.%) and 530.23 eV (26.76 at.%) are the dominant peaks corresponding to the Co−OC and the CoOCo bonds, respectively, suggesting that the C groups of g–C_3_N_4_ serve as the nucleation and anchoring sites for Co_3_O_4_ [78]. The binding energies of O 1s peaks at 531.26 eV and 532.53 are attributed to adsorbed oxygen species (water, oxygen, CO_2_) [58,59,66,80]. In Figure 6a, the high-resolution XPS spectrum of Co 2p presents dominant peaks at 779.9 eV (2p_3/2_) and 795.5 eV (2p_1/2_), which are accompanied by shake-up satellite peaks at approximately 790.4 eV and 805.7 eV, respectively. The results show the occurrence of high-spin Co oxide species, indicating an admixture of Co^2+^ and Co^3+^, the two cations present in the spinel structure of Co_3_O_4_ [64,78,80,81]. The Co 2p_3/2_ and Co 2p_1/2_ peaks of the Co species at 779.9 and 795.5 eV, giving a spin-energy separation of 15.6 eV, is the characteristic of a Co_3_O_4_ phase and matches well with the reported data [82,83,84]. Meanwhile, the deconvolution of the XPS spectrum of Co 2p_3/2_ confirms the presence of different oxidation states of Co atoms as Co_3_O_4_ (779.48 eV), and Co(II) oxides/hydroxides, including possible Co–N_x_ structures, CoO, CoOOH, Co(OH)_2_ (780.79 eV, 782.39 eV), with overlapping binding energies of different oxide and hydroxide forms [64,77]. In addition, the peak with a binding energy of 779.48 eV, attributed to the Co_3_O_4_ species, clearly dominates over the other identified peaks, accounting for 40.22 at.%, thus confirming the successful synthesis of Co_3_O_4_ giving rise to the formation of a hybrid nanostructure with g–C_3_N_4_ within the prepared Co_3_O_4_/gCN catalyst.

In the case of Co/gCN-MWS and Co/gCN-HTS catalysts, the XPS data for Co 2p show the peak shift to the region of higher binding energy, indicating the disappearance of Co_3_O_4_ species and the predominance of different forms of Co(II)-based substances (Figure 6b,c). At the same time, the XPS data for O 1s are also detected at higher binding energy values of 531.12, 532.27, and 533.17 eV for the Co/gCN-MWS catalyst and 531.45, 532.28, and 533.25 eV for the Co/gCN-HTS catalyst, corresponding to adsorbed oxygen species (Figure 5b,c). In contrast, the N 1s signal for these catalysts is located almost in the same binding energy region of approximately 398–401 eV for the Co_3_O_4_/gCN catalyst, but the intensities of the deconvoluted peaks differ considerably (Figure 4b,c). The above results have shown that both Co_3_O_4_ and g–C_3_N_4_ were successfully synthesized to form a hybrid nanostructure in the prepared Co_3_O_4_/gCN catalyst, whereas, in the case of Co/gCN-MWS and Co/gCN-HTS catalysts, the Co(II)-based substances were dominant.

**Figure 7:**
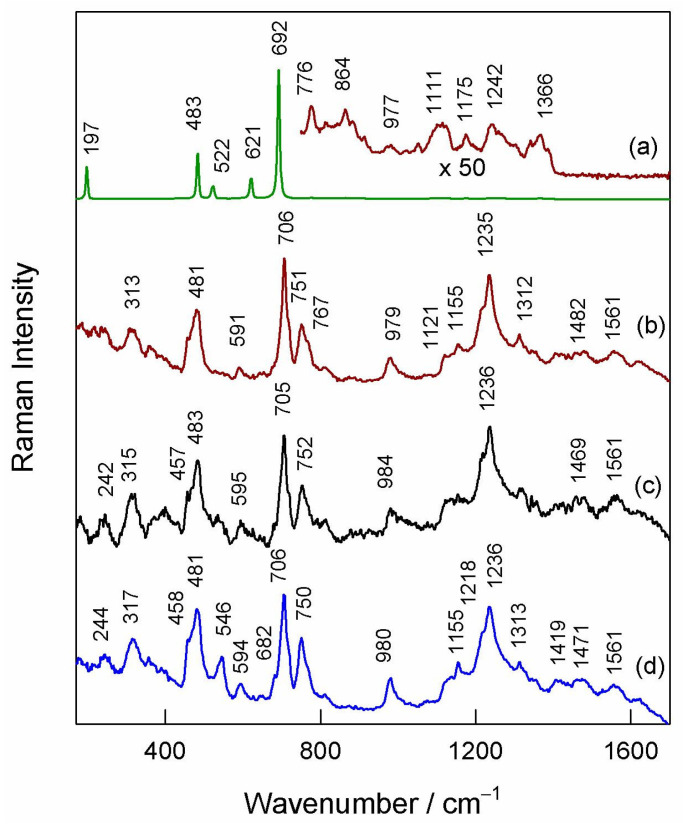
Raman spectra of samples: (**a**) Co_3_O_4_/gCN, (**b**) Co/gCN-HTS, (**c**) Co/gCN-MWS), and (**d**) gCN. Spectra are normalized to the intensity of the most intense band. The excitation wavelength is 830 nm (1.7 mW).

Figure 7 compares 830 nm excited Raman spectra of differently prepared Co/gCN samples and graphitic carbon nitride. The Raman spectrum from a sample prepared by thermal treatment of both Co(NO_3_)_2_ and melamine (Co_3_O_4_/gCN) shows intense and well-defined bands at 197, 483, 522, 621, and 692 cm^−1^ assigned to the *F*^(3)^_2g_, *E*_g_, *F*^(2)^_2g_, *F*^(1)^_2g_, and *A*_1g_ Raman active modes of the spinel Co_3_O_4_ structure, respectively [85,86,87]. The peak position of the *A*_1g_ mode coincides well with the high-quality crystal structure [87]. It was demonstrated that defective structure results in a shift of this mode to lower wavenumbers [87,88]. The presence of the gCN matrix can be visible from very-low-intensity bands in the frequency region from 700 to 1400 cm^−1^ [89,90,91]. The Raman spectra from other samples differ considerably compared with Co_3_O_4_/gCN (Figure 7).

No bands characteristic of crystalline Co_3_O_4_ are visible in these spectra; all observed features can be assigned to vibrations of the gC_3_N_4_ structure [89,90,91]. Thus, two strong bands located at 706 and 1235 cm^−1^ were assigned to the breathing vibrations of the s-triazine ring [90]. The low-intensity feature at 1561 cm^−1^ might be related to the graphitic G band, while the low-intensity band near 1469–1482 cm^−1^ might be related to the presence of amorphous carbon. It should be noted that the Raman spectrum of the gC_3_N_4_ matrix in the case of sample Co_3_O_4_/gCN is different compared with the other three structures. This might be related to the modification of graphitic carbon nitride structure because of the presence of the Co_3_O_4_ phase. The absence of Co_3_O_4_ bands in the spectra of samples prepared by microwave-assisted and hydrothermal-assisted methods suggests that the structure of cobalt oxides is amorphous.

### 3.2. Investigation of Electrocatalyst Activity for HER

**Figure 8:**
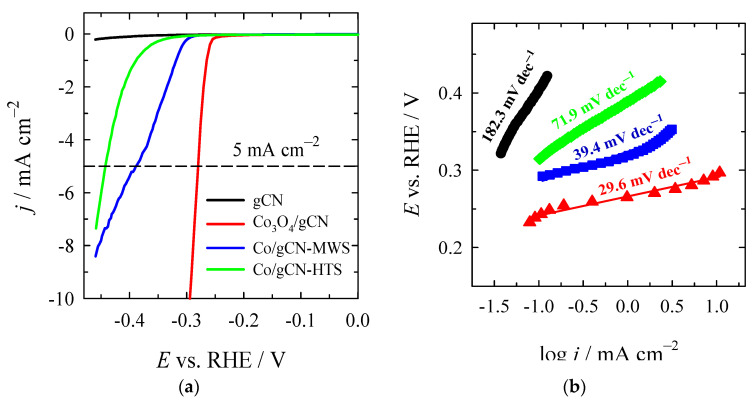
(**a**) HER polarization curves of gCN, Co_3_O_4_/gCN, Co/gCN-MWS, and Co/gCN-HTS catalysts in 1 M KOH solution at a potential scan rate of 2 mV s^−1^; (**b**) The corresponding Tafel slopes for each catalyst.

The electrocatalytic activity of synthesized catalysts was investigated for HER and OER in an alkaline medium. The HER polarization curves recorded on the investigated Co_3_O_4_/gCN, Co/gCN-MWS, and Co/gCN-HTS catalysts in alkaline media are shown in Figure 8a, whereas data of the electrochemical performance of the tested catalysts are given in Table 2.

As seen, the lowest onset potential (*E*_onset_) of −0.24 V for the HER exhibits the Co_3_O_4_/gCN catalyst compared with Co/gCN-MWS, Co/gCN-HTS, and gCN catalysts (Table 2). Additionally, the latter catalyst shows a significantly higher current density and the lowest overpotential of −280.5 mV for the HER to reach a current density of 5 mA cm^−2^ (η_5_) (Figure 8a), followed by Co/gCN-MWS (−389.4 mV) and Co/gCN-HTS (−444.1 mV). The overpotential value at a current density of 10 mA·cm^−2^ (η_10_) for Co_3_O_4_/gCN was found to be −294.1 mV (Figure 8a).

The reaction kinetics and mechanism of the as-prepared catalysts can be evaluated based on Tafel slopes determined from the following equation (Equation (5)) [76]:

*η* = b · log *j*/*j*_0_ (5)

*η* is the overpotential, b is the Tafel slope, *j* is the experimental current density, and *j*_0_ is the exchange current density. The plot of *η* versus log *j* represents the Tafel slope. It is widely accepted that HER proceeds by either the Volmer–Heyrovsky or Volmer–Tafel mechanisms, and in alkaline media, it involves three main steps, as shown in Equations (6) to (8) [63]:

* + H_2_O + e^−^ ↔ *H_ads_ + OH^−^  (Volmer step) (6)

*H_ads_ + e^−^ + H_2_O ↔ H_2_ + OH^−^ + *  (Heyrovsky step) (7)

2*H_ads_ ↔ H_2_ + *       (Tafel step) (8)

H_ads_ denotes the H_2_ adsorbed to the metal sites, where * represents the metal sites. The theoretical Tafel slopes in the aforementioned reaction steps are 120 mV dec^−1^, 40 mV dec^−1^, and 30 mV dec^−1^, respectively. Figure 8b shows the Tafel slopes of gCN, Co_3_O_4_/gCN, Co/gCN-MWS, and Co/gCN-HTS catalysts, pointing to the rate-determining step and the likely mechanism associated with electrocatalytic hydrogen generation. The Co_3_O_4_/gCN catalyst was found to have the lowest Tafel slope of 29.6 mV dec^−1^ compared to Co/gCN-MWS (39.4 mV dec^−1^), Co/gCN-HTS (71.9 mV dec^−1^), and gCN (182.3 mV dec^−1^). This predicts the favorable HER kinetics following the Volmer–Tafel mechanism on the Co_3_O_4_/gCN catalyst, suggesting that the Tafel recombination step is the dominating process, indicating that the rapid charge transfer process is present. In the case of Co/gCN-MWS and Co/gCN-HTS materials, the Heyrovsky reaction is the rate-determining step, and the Volmer–Heyrovsky reaction is expected to take place. Likely, the lower performance of Co/gCN-MWS and Co/gCN-HTS towards HER, as compared to Co_3_O_4_/gCN, is related to the poor adsorption of H^+^ ions onto the catalyst surface.

Among the investigated catalysts, the lower *E*_onset_ of −0.24 V, a small overpotential of −280.5 mV at 5 mA cm^−2^, and a low Tafel slope of 29.6 mV dec^−1^ of Co_3_O_4_/gCN indicate that this catalyst prepared by annealing Co(NO_3_)_2_ together with melamine affords the fabrication of efficient catalyst with the highest HER activity in an alkaline medium.

### 3.3. Investigation of Electrocatalyst Activity for OER

**Figure 9:**
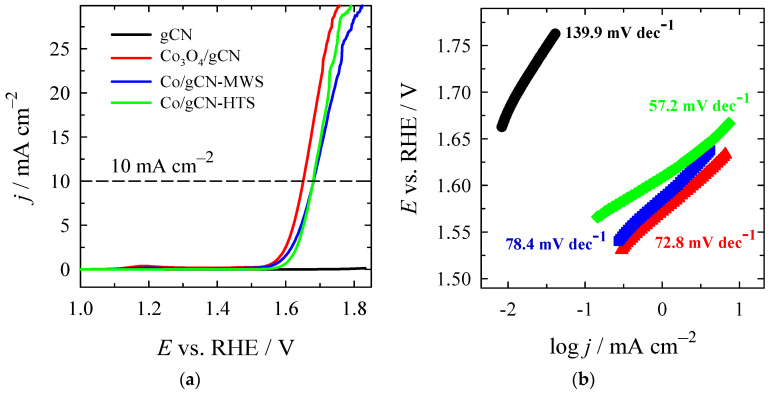
(**a**) OER polarization curves of gCN, Co_3_O_4_/gCN, Co/gCN-MWS, and Co/gCN-HTS catalysts in N_2_-saturated 1 M KOH solution at a potential scan rate of 2 mV s^−1^; (**b**) The corresponding Tafel slopes for each catalyst.

The activity of catalysts for OER was further evaluated. The obtained data are presented in Figure 9 and Table 3. Figure 9a,b illustrates the OER polarization curves and the corresponding Tafel slopes recorded on the gCN, Co_3_O_4_/gCN, Co/gCN-MWS, and Co/gCN-HTS at a slow scan rate of 2 mV s^−1^ in N_2_-saturated 1 M KOH solution. The summarized data are also given in Table 3.

Bare gCN shows poor activity for OER with a low current density, even at high overpotential. On the contrary, all of the Co-modified gCNs gave much higher current densities and lower overpotentials than gCN, meaning a significant improvement in catalytic activity toward OER. *E*_onset_ values were found in a gradually increasing order, as follows: Co_3_O_4_/gCN (1.5162 V) < Co/gCN-MWS (1.5230 V) < Co/gCN-HTS (1.5394 V) < gCN (1.6404 V), with overpotential values of 286.2, 293.0, 309.4, and 410.4 mV, respectively (Table 3). Overpotentials to achieve the current density of 10 mA·cm^−2^ were found as 422.3, 451.3, and 450.9 mV for Co_3_O_4_/gCN, Co/gCN-MWS, and Co/gCN-HTS, respectively (Table 3). The Tafel slope of Co/gCN-HTS (57.2 mV dec^−1^) is lower than those of Co_3_O_4_/gCN and Co/gCN-MWS (Figure 9b, Table 3), indicating better catalytic activity for the OER. A 4e^−^ mechanism is widely accepted for the OER process. The steps of the reaction in alkaline media can be represented by Equations (9)–(12) [29,65,92]:

* + OH^−^ ↔ e^−^ + OH* (9)

OH^−^ + OH* ↔ e^−^ + O* + H_2_O (10)

OH^−^ + O* ↔ e^−^ + OOH* (11)

OH^−^ + OOH* ↔ H_2_O +*+ e^−^ + O_2_ (12)

where * denotes the electrocatalyst’s adsorption site; similarly, during OER, the adsorbed intermediates are OH*, O*, and OOH*. The first step of the OER process, denoted by Equation (9), is the electrosorption of OH^−^ onto the active sites of the catalyst’s surface. Higher oxidation state metal species are more susceptible to adsorb OH^−^, accelerate the multielectron transportation process, and, hence can therefore enhance the OER process [29,92]. According to the XPS analysis, the synthesized catalyst Co_3_O_4_/gCN has the highest valence state of the rest prepared and is considered to be the most favorable for the adsorption of OH^−^. In addition, it is generally accepted that during the OER process, the phase transformation of Co-based materials, such as oxides (Co_3_O_4_), to hydroxides or oxyhydroxides plays a rather significant role [92]. In this view, the Co_3_O_4_/gCN catalyst with the lowest onset overpotential of 286.2 mV and the lowest overpotential of 422.3 mV reaches 10 mA cm^−2^ with a Tafel slope of 72.8 mV dec^−1^. This is predicted to have the most perfect OER reaction kinetics compared to the other catalysts studied in the current work. The electrocatalytic behavior of the Co_3_O_4_/gCN catalyst and those obtained by microwave-assisted and hydrothermal syntheses are also compared with previously reported works and are presented in Table 4. These overpotentials are comparable to those previously reported for state-of-the-art non-precious metal catalysts for WS in alkaline media.

The above results demonstrate that Co_3_O_4_/gCN could act as a bifunctional catalyst due to the notable activity towards HER and OER, and for total WS in practical applications, it is a promising alternative to noble metal-based electrocatalysts. The potential to produce the Co_3_O_4_/gCN catalyst with a high crystallinity of high-purity Co_3_O_4_ using a simple annealing procedure contributes to significantly improved HER and OER performances. Appropriate incorporation of Co species into the tri-s-triazine moieties creates unique active redox sites of Co–(N)–C for pronounced HER and OER activities.

### 3.4. Determination of ECSAs of Electrocatalysts

**Figure 10:**
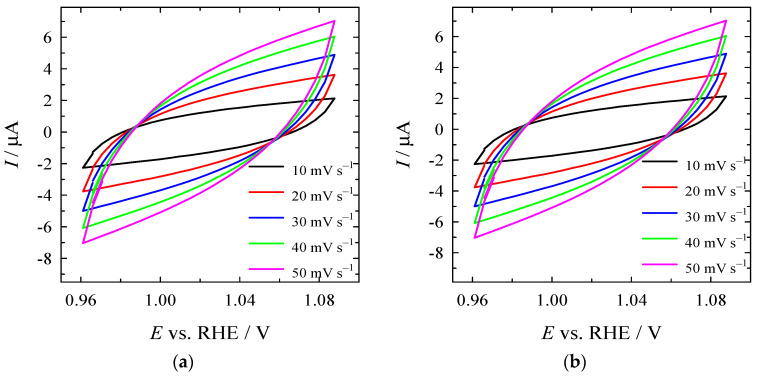

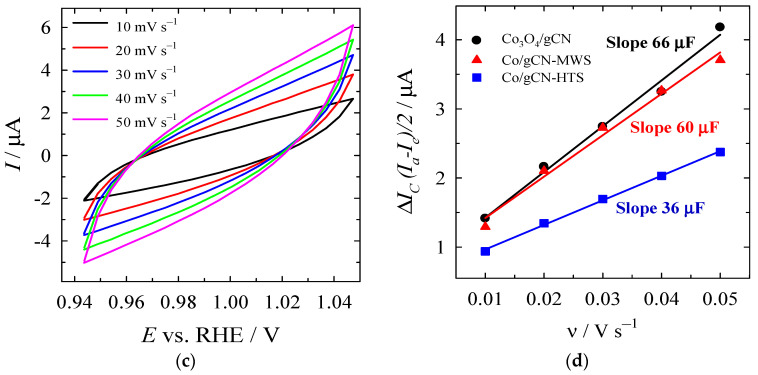
CVs of (**a**) Co_3_O_4_/gCN, (**b**) Co/gCN-MWS, and (**c**) Co/gCN-HTS in N_2_-saturated 1 M KOH in the non-faradaic potential region at different scan rates. (**d**) Capacitive current as a function of scan rate.

For the determination of ECSA, the C_dl_ values were determined from CV curves recorded at various scan rates under the non-faradic region (Figure 10a–c), followed by the calculation of the slope of the curve obtained by plotting the difference in anodic and cathodic current against the scan rate (Figure 10d). ECSA values were calculated by normalizing the C_dl_ to the specific capacitance (40 μF cm^−2^). C_dl_ was found to be 66, 60, and 36 µF for Co_3_O_4_/gCN, Co/gCN-MWS, and Co/gCN-HTS (Figure 10d), whereas the calculated ECSA values were 1.65, 1.5, and 0.9 cm^2^ for Co_3_O_4_/gCN, Co/gCN-MWs, and Co/gCN-HTS, respectively. The higher ECSA of Co_3_O_4_/gCN than Co/gCN-MWS and Co/gCN-HTS represents the higher number of active sites and, consequently, larger electrocatalytic activity of this catalyst for HER and OER.

**Figure 11:**
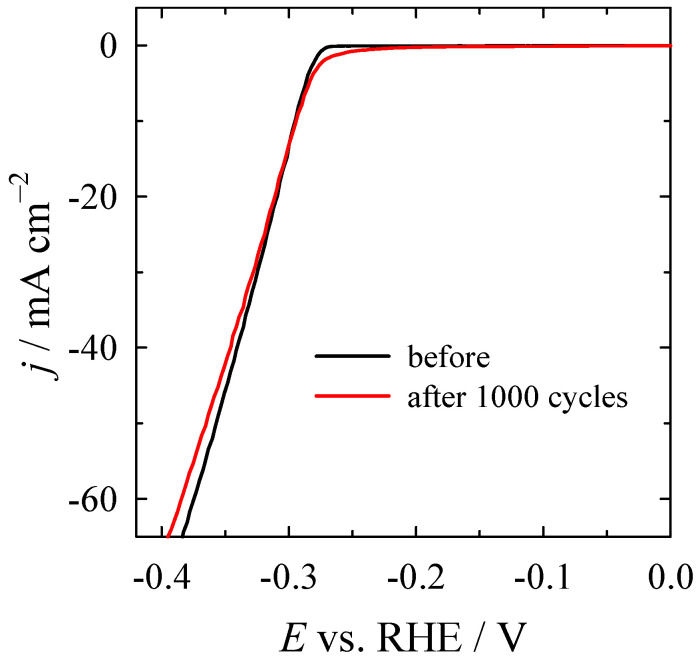
LSV curves before and after 1000 cycles for HER for Co_3_O_4_/gCN recorded at 2 mV s^−1^ at 1600 rpm.

Additionally, the stability of the Co_3_O_4_/gCN catalyst was tested for 1000 cycles at a constant scan rate of 100 mV s^−1^ at a rotation rate of 1600 rpm in N_2_-saturated 1 M KOH. Figure 11 presents the LSVs before and after 1000 cycles recorded at 2 mV s^−1^ at 1600 rpm. The LSV curve exhibited almost no differences from the initial test before the CV cycles (Figure 11) at a constant current density of 10 mA cm^−2^, indicating high stability for HER and maintaining the same morphology and structure after the HER test.

### 3.5. EIS Measurements

**Figure 12:**
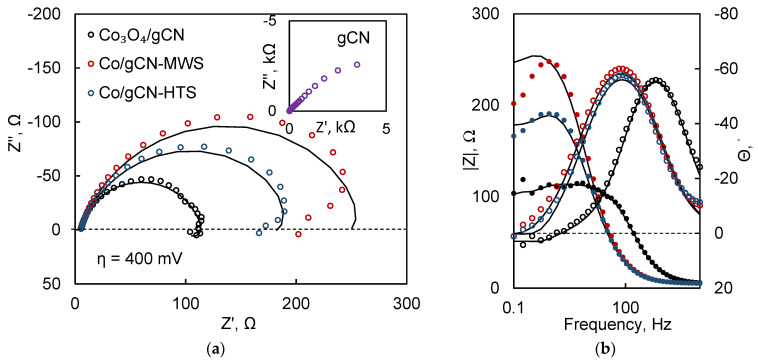
EIS spectra of catalytic and gCN films, obtained at 400 mV overpotential in Nyquist (**a**) and Bode (**b**) coordinates. Solid lines show fits to the equivalent circuit in Figure 13.

EIS was used to obtain more detailed information on the interfacial kinetics of these catalysts. A relatively large overpotential (400 mV) was chosen so that the spectral response would be indicative of the catalytic Faradaic reactions rather than any capacitance-dominated processes. From the profile of the Nyquist plots (Figure 12a), it is apparent that the impedance magnitude is smallest for the Co_3_O_4_/gCN film, corresponding well to LSV data (Figure 8a). The Co/gCN-MWS and HTS catalysts perform slightly worse, which is indicated by their larger impedances at the same overpotential. Bare gCN is included for comparison in the inset of Figure 12a and, at the same conditions, shows virtually no charge transfer activity.

Another feature of the spectra is the emerging low-frequency inductive loops, particularly apparent for the Co_3_O_4_/gCN sample. This phenomenon is said to occur due to the slow relaxation of adsorbed reaction intermediates and characterizes a mechanism with two electrochemical steps [97,98]. This process can be either HER or ORR, but EIS does not allow us to distinguish them without additional experimentation. The Bode plots (Figure 12b) also reveal an interesting feature: for the Co_3_O_4_/gCN catalyst, the phase maximum emerges at significantly higher frequencies (~1 kHz). This means that the current responds more swiftly to potential perturbation, and the catalytic reaction occurs at a faster rate.

**Figure 13:**
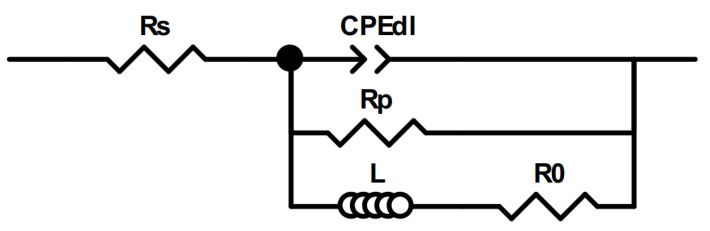
The equivalent electric circuit used to fit EIS data.

Equivalent circuit fitting was carried out to gain quantitative data on the performance of these catalysts. To fit a spectrum with an inductive loop with no mass transport limitations, typically, an equivalent circuit, as shown in Figure 13, is used [99].

In this circuit, R_s_ is the solution resistance, CPE_dl_ is a constant phase element that accounts for the double layer capacitance of an inhomogeneous surface; R_p_ is the resistance of the Faradaic process, here approximated as the charge transfer resistance; and L and R_0_ account for the low-frequency inductance.

CPE values were recalculated into capacitance by Equation (13) [99], and the results are shown in Table 5.

(13) $$C^{\varphi }=\frac{Q}{{\left(R^{-1}\right)}^{1-\varphi }}$$

The values of the low-frequency resistance R_p_ correspond most directly to the rate-determining step of the catalytic reaction, and the lowest value (109.5 Ω) is calculated for Co_3_O_4_/gCN. The inductive elements (L, R_0_) occur due to the slow adsorption of reaction intermediates on the surface of the catalyst and the delay between coverage and removal during potential modulation [99]. Therefore, lower equivalent circuit element values mean faster surface relaxation [100], and the Co_3_O_4_/gCN film potentially has the fastest adsorbate relaxation kinetics.

## 4. Conclusions

Cobalt-particle-supported graphitic carbon nitride catalysts were obtained using three different methods: materials annealing, microwave-assisted, and hydrothermal syntheses. It was found that the catalyst prepared via the annealing of Co(NO_3_)_2_ with melamine at 520 °C exhibited the highest activity for hydrogen evolution compared to the other Co-supported gCN catalysts prepared via microwave-assisted and hydrothermal syntheses. The Co_3_O_4_/gCN catalyst shows the highest current density and gives the lowest overpotential of −280.5 mV for HER to reach a current density of 5 mA cm^−2^ in 1 M KOH. Overpotentials to reach the current density of 5 mA·cm^−2^ for HER were found in a gradually increasing order, as follows: Co_3_O_4_/gCN (−280.5 mV) < Co/gCN-MWS (−389.4 mV) < Co/gCN-HTS (−444.1 mV). Overpotentials to reach the current density of 10 mA·cm^−2^ for OER are in a gradually increasing order, as follows: Co_3_O_4_/gCN (422.3 mV) < Co/gCN-HTS (450.9 mV) < Co/gCN-MWS (451.3 mV).

Thermal annealing of both Co(NO_3_)_2_ and melamine at 520 °C for 4 h results in the preparation of an efficient Co_3_O_4_/gCN catalyst for the HER with the lower *E*_onset_ of −0.24 V, a small overpotential of −294.1 mV at −10 mA cm^−2^, and a low Tafel slope of −29.6 mV dec^−1^. Additionally, it has the lowest onset overpotential of 286.2 mV and the lowest overpotential of 422.3 mV to reach a current density of 10 mA cm^−2^ with the Tafel slope of 72.8 mV dec^−1^ for OER compared to Co/gCN-MWS and Co/gCN-HTS. Importantly, the annealing treatment of Co(NO_3_)_2_ with melamine to obtain the Co_3_O_4_/gCN catalyst is a potential method for its practical applications in electrochemical water splitting.

EIS analysis of the films revealed that the Co_3_O_4_/gCN film has the fastest adsorbate relaxation kinetics, which is likely one reason for its improved electrocatalytic activity.

## Figures and Tables

**Table 1:** The elemental composition of the tested catalysts.

Sample	C1s		N1s		O1s		Co2p	
	BE, eV	at.%	BE, eV	at.%	BE, eV	at.%	BE, eV	at.%
gCN	284.95	10.55	398.86	67.92	530.89	12.04		
	286.60	6.10	400.07	16.03	532.49	57.24	−	−
	288.31	64.66	401.28	10.59	533.42	30.72		
	288.70	18.68	404.66	5.46				
Co_3_O_4_/gCN	284.42	13.42	398.27	33.26	529.64	36.81	779.48	40.22
	285.08	36.44	399.33	42.74	530.23	26.76	780.79	38.74
	286.36	32.62	400.78	24.00	531.26	21.49	782.39	21.04
	287.68	17.52			532.53	14.95		
Co/gCN-MWS	284.47	9.98	398.56	62.25	531.12	42.40	780.87	49.74
	285.06	17.70	399.22	28.25	532.27	34.61	782.79	30.61
	286.32	19.26	400.81	9.50	533.17	23.00	786.52	19.66
	288.20	53.06						
Co/gCN-HTS	285.04	22.19	397.73	57.24	531.45	50.85	781.68	56.85
	286.30	28.25	398.93	28.42	532.28	32.47	784.05	23.90
	287.44	35.03	400.21	14.35	533.25	16.69	787.43	19.26
	288.72	14.53						

**Table 2:** Electrochemical performance of the tested catalysts toward HER in alkaline media.

Catalysts	*E*_onset_, V at *j* = −0.1 mA cm^−2^	η_5_ *, mV	η_10_ **, mV	Tafel Slope, mV dec^−1^
gCN	−0.40	−	−	182.3
Co_3_O_4_/gCN	−0.24	−280.5	−294.1	29.6
Co/gCN-MWS	−0.29	−389.4	−	39.4
Co/gCN-HTS	−0.31	−444.1	−	71.9

* Overpotential at 5 mA cm^−2^. ** Overpotential at 10 mA cm^−2^.

**Table 3:** Electrochemical performance of the tested catalysts toward OER in alkaline media.

Catalysts	*E*_onset_, V at *j* = 0.1 mA cm^−2^	η_onset_, mV	*E*, V at *j* = 10 mA cm^−2^	η_10_ **, mV	Tafel slope, mV dec^−1^
gCN	1.6404	410.4	−	−	139.9
Co_3_O_4_/gCN	1.5162	286.2	1.6523	422.3	72.8
Co/gCN-MWS	1.5230	293.0	1.6813	451.3	78.4
Co/gCN-HTS	1.5394	309.4	1.6809	450.9	57.2

** Overpotential at 10 mA cm^−2^.

**Table 4:** Electrochemical parameters of different Co-based gCN catalysts.

Catalysts	Electrolyte	HER		OER		Ref.
		η_10_ *, mV	Tafel Slope, mV dec^−1^	η_10_ *, mV	Tafel SLOPE, mV dec^−1^	
(GCN)50	1 M KOH	160	90.76	310	49.86	[63]
C_3_N_4_@Co(OH)_2_ + PA	1 M KOH	–	–	329	199	[64]
CGD	1 M KOH	287	94	445	69	[65]
CoOx	1 M KOH	–	–	423	42	[93]
CoNi_2_S_4_/gCN	1 M KOH	160	90.76	310 at 30 mA cm^−2^	49.86	[63]
g–C_3_N_4_/Co_3_O_4_/ZnS QDs	1 M KOH	304	71	–	–	[62]
Co_2_SnO_4_/g–C_3_N_4_	1 M KOH	41	48	260	47	[94]
Co-SCN/RGO	1 M KOH	150	94	250	96	[95]
Co_3_O_4_/g–C_3_N_4_	1 M KOH	313	169	315	67	[96]
Co_3_O_4_MoO_3_/g-C_3_N_4_	1 M KOH	125	94	206	60	[96]
Co_3_O_4_/gCN	1 M KOH	294.1	29.6	422.3	72.8	This work
Co/gCN-MWS	1 M KOH	–	39.4	451.3	78.4	This work
Co/gCN-HTS	1 M KOH	–	71.9	450.9	57.2	This work

* Overpotential at 10 mA cm^−2^.

**Table 5:** Equivalent circuit fitting data of catalytic films.

Catalyst	R_s,_ Ω	C, mF	R_p,_ Ω	L	R_0,_ Ω
Co_3_O_4_/gCN	5.03	6.45 × 10^−5^	109.5	574.1	622.9
Co/gCN-HTS	5.06	6.13 × 10^−4^	201.4	408.7	1266
Co/gCN-MWS	5.03	5.93 × 10^−4^	263.6	2045	2730

## Data Availability

Not applicable.
